# Risk factors for SARS-CoV-2 seropositivity in a health care worker population during the early pandemic

**DOI:** 10.1186/s12879-023-08284-y

**Published:** 2023-05-16

**Authors:** Sebastian D. Schubl, Cesar Figueroa, Anton M. Palma, Rafael R. de Assis, Aarti Jain, Rie Nakajima, Algimantas Jasinskas, Danielle Brabender, Sina Hosseinian, Ariana Naaseh, Oscar Hernandez Dominguez, Ava Runge, Shannon Skochko, Justine Chinn, Adam J. Kelsey, Kieu T. Lai, Weian Zhao, Peter Horvath, Delia Tifrea, Areg Grigorian, Abran Gonzales, Suzanne Adelsohn, Frank Zaldivar, Robert Edwards, Alpesh N. Amin, Michael J. Stamos, Philip S. Barie, Philip L. Felgner, Saahir Khan

**Affiliations:** 1 Department of Surgery, School of Medicine, University of California Irvine, Irvine, CA USA; 2 Institute for Clinical and Translational Sciences, University of California Irvine, Irvine, CA USA; 3 Department of Physiology and Biophysics, University of California Irvine, Irvine, CA USA; 4 School of Medicine, University of California Irvine, Irvine, CA USA; 5 Department of Pharmaceutical Sciences, School of Medicine, University of California Irvine, Irvine, CA USA; 6 Department of Pathology, School of Medicine, University of California Irvine, Irvine, CA USA; 7 Department of Surgery, Keck School of Medicine, University of Southern California, Los Angeles, CA USA; 8 Department of Medicine, School of Medicine, University of California Irvine, Irvine, CA USA; 9 Department of Surgery, Weill Cornell Medicine, New York, NY USA; 10 Division of Infectious Diseases, Department of Medicine, Keck School of Medicine, University of Southern California, 1520 San Pablo St., Los Angeles, CA 90033 USA

**Keywords:** SARS-CoV-2, Risk analysis, Healthcare workers, Serology

## Abstract

**Background:**

While others have reported severe acute respiratory syndrome-related coronavirus 2(SARS-CoV-2) seroprevalence studies in health care workers (HCWs), we leverage the use of a highly sensitive coronavirus antigen microarray to identify a group of seropositive health care workers who were missed by daily symptom screening that was instituted prior to any epidemiologically significant local outbreak. Given that most health care facilities rely on daily symptom screening as the primary method to identify SARS-CoV-2 among health care workers, here, we aim to determine how demographic, occupational, and clinical variables influence SARS-CoV-2 seropositivity among health care workers.

**Methods:**

We designed a cross-sectional survey of HCWs for SARS-CoV-2 seropositivity conducted from May 15th to June 30th 2020 at a 418-bed academic hospital in Orange County, California. From an eligible population of 5,349 HCWs, study participants were recruited in two ways: an open cohort, and a targeted cohort. The open cohort was open to anyone, whereas the targeted cohort that recruited HCWs previously screened for COVID-19 or work in high-risk units. A total of 1,557 HCWs completed the survey and provided specimens, including 1,044 in the open cohort and 513 in the targeted cohort. Demographic, occupational, and clinical variables were surveyed electronically. SARS-CoV-2 seropositivity was assessed using a coronavirus antigen microarray (CoVAM), which measures antibodies against eleven viral antigens to identify prior infection with 98% specificity and 93% sensitivity.

**Results:**

Among tested HCWs (n = 1,557), SARS-CoV-2 seropositivity was 10.8%, and risk factors included male gender (OR 1.48, 95% CI 1.05–2.06), exposure to COVID-19 outside of work (2.29, 1.14–4.29), working in food or environmental services (4.85, 1.51–14.85), and working in COVID-19 units (ICU: 2.28, 1.29–3.96; ward: 1.59, 1.01–2.48). Amongst 1,103 HCWs not previously screened, seropositivity was 8.0%, and additional risk factors included younger age (1.57, 1.00-2.45) and working in administration (2.69, 1.10–7.10).

**Conclusion:**

SARS-CoV-2 seropositivity is significantly higher than reported case counts even among HCWs who are meticulously screened. Seropositive HCWs missed by screening were more likely to be younger, work outside direct patient care, or have exposure outside of work.

**Supplementary Information:**

The online version contains supplementary material available at 10.1186/s12879-023-08284-y.

## Background

Protecting health care workers (HCWs) during the coronavirus disease 2019 (COVID-19) pandemic is essential to mounting an effective response, as outbreaks among this population could potentially cripple health care delivery. Current case identification relies on symptom and temperature screening with follow-up testing by severe acute respiratory syndrome coronavirus-2 (SARS-CoV-2) quantitative reverse transcriptase-polymerase chain reaction (qRT-PCR). This approach underestimates disease prevalence by missing cases of asymptomatic infection and false negatives due to suboptimal timing, flawed specimen collection, or low viral load [1, 2]. Given the importance of asymptomatic persons in the transmission of SARS-CoV-2, estimated to account for 59% of overall transmission, including 24% from asymptomatic persons and 35% from pre-symptomatic persons [3], identification of risk factors that may augment identification of asymptomatic infection in HCWs is crucial to protecting patients and the health care system [1].

Serologic testing can help to determine the true prevalence of COVID-19 by identifying previously infected persons who had minimal symptoms so were missed by the current testing paradigm [4, 5]. Multiple COVID-19 seroprevalence studies have been performed in different populations but are limited by low specificity in low-prevalence populations or potential selection bias from the use of convenience sampling, with estimated seroprevalence of comparable populations during the study period ranging from 1.0 to 11.2% [6–11]. Estimated seroprevalence among HCW varies widely, with some studies finding similar or even lower prevalence compared to the surrounding community, but most studies noting a significant proportion of asymptomatic infections [8, 12–18].

Risk factors for SARS-CoV-2 infection amongst HCW were initially extrapolated from studies of hospitalized patients with severe disease that may not be generalizable to the population at large [19]. Previous studies to identify risk factors amongst HCWs were performed in early outbreak setting prior to current infection control practices so may not be currently applicable [20, 21]. More recent studies have conflicting results as to whether occupational exposures confer an increased risk of SARS-CoV-2 seropositivity and may be limited by suboptimal performance of the assays upon external validation, heterogeneity in the study populations, and lack of control for confounding due to the use of univariate analyses [22–25].

This study measured SARS-CoV-2 seropositivity amongst 1,557 HCWs at the University of California-Irvine Health, a 418-bed academic medical center in Orange County, California, from May 15th to June 30th 2020, using a novel coronavirus antigen microarray (CoVAM). This CoVAM utilizes 11 SARS-CoV-2 IgG and IgM antigens to determine prior infection with 98% specificity and 93% sensitivity based on validation in 91 rt-PCR-positive cases and 88 pre-pandemic negative controls. The CoVAM also is used to distinguish SARS_CoV-2 infection from prior infection with other human coronaviruses [26]. This performance and level of validation compares favorably to other serologic assays based on a single antigen [27, 28], and the predictive model based on multiple antigens outperformed models based on any single antigen during validation of the COVAM [26]. A multivariable analysis was used to probe associations among demographic, clinical, and occupational risk factors and SARS-CoV-2 seropositivity among HCWs.

## Methods

### Study design, setting, and population

The study, a prospective cohort study, with the first time point presented here as a cross-sectional analysis, was approved by the Institutional Review Board of the University of California-Irvine under Protocol HS 2020–5818. All 5,349 employees who worked in the hospital were eligible. Universal daily symptom and temperature screening was initiated on April 14, 2020, with subsequent immediate rt-PCR testing for any HCW with symptoms or fever or disclosure of a confirmed or suspected COVID-19 contact. A primary study site in the main hospital building of University of California Irvine Health (Orange, California, USA) was open from May 15 to May 29, 2020 to all employees who provided electronic consent (open enrollment cohort). In addition, all employees who had been tested for SARS-CoV-2 by rt-PCR at the primary study site due to symptoms or possible exposure, or who provided direct patient care in COVID-19 clinical units or similar control units, were invited via email and provided electronic consent to participate at a secondary study open from May 15 to June 30, 2020 (targeted enrollment cohort). This second cohort was included to enrich the study for HCW with COVID-19 infection, symptoms, or exposure.

### Study procedures

Participants were given a unique study identifier and a mobile phone link to a Research Electronic Data Capture (REDCap, Vanderbilt University, Nashville, TN) survey to collect data on demographic, clinical, and occupational risk factors (Supplementary Fig. 2 ). At the primary study site, participants then underwent capillary blood collection via fingerstick using a disposable lancet into microfuge capillary tubes (BD Microtainer). After centrifugation at 1500 x g for 10 min, supernatant plasma was collected, frozen, and transported for laboratory analysis (for testing for SARS-CoV-2 antibodies on the CoVAM). At the secondary study site, participants underwent phlebotomy into gold-top tubes (BD Biosciences, San Jose, CA) for centrifugation and collection of serum, from which an aliquot was frozen at 0 C° and transported for laboratory analysis (for testing for SARS-CoV-2 antibodies on the CoVAM). All specimens were labeled with unique identifiers accessible only via a secure key.

### Laboratory assay

The CoVAM includes 67 antigens from respiratory viruses, including 11 antigens from SARS-CoV-2 (Sino Biological U.S. Inc., Wayne, PA). Antigens were printed onto microarrays in quadruplicate, probed with serum specimens and secondary antibodies for IgM and IgG, and imaged to determine background-subtracted median fluorescence intensity [29–31]. Briefly, CoVAM data for each specimen were compared with 91 rt-PCR-positive cases with blood collected ≥ 7 days (range 7–50, median 11) post-symptom onset and 88 pre-pandemic controls with blood collected prior to November 1, 2019, which were split randomly into 70% training set and 30% testing set for model development. Based on IgM and IgG antibodies against the 11 SARS-CoV-2 antigens on the array, a logistic regression model was trained on positive and negative controls in the training set to determine optimal weighted combinations of reactive antigens to calculate composite SARS-CoV-2 antibody titers that discriminate the two groups, with reactivity thresholds selected to achieve maximum sensitivity while maintaining ≥ 98% specificity. The model was tested on the testing set and achieved 92.7% sensitivity and 97.7% specificity for detecting prior SARS-CoV-2 infection based on composite IgM or IgG positivity [26]. A detailed description of the development and validation of the CoVAM has been previously published [26].

### Statistical analysis

The prevalence of SARS-CoV-2 seropositivity was calculated in the study population as the proportion of HCWs who were classified as seropositive, and the prevalence of SARS-CoV-2 seropositivity was calculated within categories of each demographic, clinical and occupational risk factor and compared to the study population by expressing as odds ratio (OR) with 95% confidence interval. In order to assess the associations between clinical and occupational risk factors and seropositivity, multivariable logistic regression models were constructed to control for potential confounding due to demographic and health-related factors associated with both occupational exposure and underlying risk for seropositivity; potential covariates were chosen by the authors based on known association with COVID-19 epidemiology and relevance to occupational health. We included in the model demographic variables (age, gender, and race/ethnicity) and health-related covariates (asthma or chronic obstructive pulmonary disease, diabetes mellitus, hypertension, self-reported smoking or vaping) which have known associations with COVID-19 epidemiology [32], in addition to known COVID-19 exposure outside of work and occupation-related variables of interest (self-reported role, location, and contact with COVID-19 patients). A targeted cohort was included in addition to the open cohort in order to gain data on healthcare workers who may be more prone to exposure- those in high-risk areas (e.g. ICU healthcare workers), a particular interest to this study. Health- and occupation-related exposures were selected based on bivariate associations with seropositivity using a p < 0.1 criterion for inclusion and added to the model in a forward stepwise regression. The final model for clinical and occupational risk factors was adjusted for age (quartiles), gender, race/ethnicity (Asian, White, Latino, Black, and Mixed/other/not reported), known COVID-19 exposure outside of work, and workplace role, location, and COVID-19 patient contact. Adjusted analyses were conducted among the entire sample and the same model was applied to the subgroup of HCWs not tested previously via rt-PCR. Model fit was evaluated using the Hosmer-Lemeshow goodness-of-fit test and C-statistic. All analyses were conducted using R software v4.0.3 (R Consortium for Statistical Computing, Vienna, Austria).

## Results

### SARS-CoV-2 seropositivity in the study population

From an eligible population of 5,349 HCWs, 1,841 (34.4%) consented to participate, including 1,108 in the open enrollment cohort and 733 in the targeted enrollment cohort. Of the targeted cohort, 343 had been tested by rt-PCR for COVID-19, 237 worked in a COVID-19 unit and 153 worked in a matched control unit of similar acuity. A total of 1,557 HCWs completed the survey and provided blood specimens to be analyzed by CoVAM, including 1,044 in the open enrollment cohort and 513 in the targeted cohort.

SARS-CoV-2 seropositivity was 10.8% in the overall HCW cohort (Table 1). Seropositivity was 17.7% amongst the 419 HCWs who had been tested by rt-PCR previously, and 8.0% among the 1,138 HCW who had not. Seropositivity in the targeted versus open enrollment cohorts matched closely the seropositivity among HCWs tested previously versus not tested by rt-PCR respectively. Of the 413 HCW tested by rt-PCR, results were available for 360 HCWs, among these 38 were PCR + and 322 PCR-, with 36 (94.7%) of the PCR + testing seropositive whereas 30 (9.3%) of the PCR- were seropositive, higher than the 8.0% seropositivity rate of those not tested by rt-PCR (Supplementary Table 4 ).

**Table 1:** Association between demographic and health-related characteristics and SARS-CoV-2 seropositivity of HCW study population and subgroups

	All HCWs (n = 1,557)			Not tested by rt-PCR (n = 1,138)			Tested by rt-PCR (n = 419)		
	HCWs, n (%)	COVID-19 AB prevalence, n (%)	OR (95% CI)^1^	HCWs, n (%)	COVID-19 AB prevalence, n (%)	OR (95% CI)^1^	HCWs, n (%)	COVID-19 AB prevalence, n (%)	OR (95% CI)^1^
**Total**	1557 (100)	165 (10.6)		1138 (100)	91 (8.0)		419 (100)	74 (17.7)	
**Age quartiles (y)**									
18–31	418 (26.8)	49 (11.7)	1.17 (0.82–1.66)	311 (27.3)	33 (10.6)	1.57 (1.00-2.45)	107 (25.5)	16 (15.0)	0.77 (0.41–1.38)
32–38	382 (24.5)	41 (10.7)	1.02 (0.69–1.47)	257 (22.6)	22 (8.6)	1.10 (0.65–1.79)	125 (29.8)	19 (15.2)	0.78 (0.43–1.36)
39–43	377 (24.2)	35 (9.3)	0.83 (0.55–1.21)	275 (24.2)	15 (5.5)	0.60 (0.33–1.03)	102 (24.3)	20 (19.6)	1.19 (0.66–2.07)
49–73	380 (24.4)	40 (10.5)	0.99 (0.67–1.43)	295 (25.9)	21 (7.1)	0.85 (0.50–1.38)	85 (20.3)	19 (22.4)	1.46 (0.80–2.59)
**Gender**									
Female	1073 (68.9)	100 (9.3)	0.66 (0.48–0.93)	781 (68.6)	57 (7.3)	0.75 (0.48–1.18)	292 (69.7)	43 (14.7)	0.53 (0.32–0.90)
Male	482 (31.0)	64 (13.3)	1.48 (1.05–2.06)	355 (31.2)	33 (9.3)	1.28 (0.81–1.99)	127 (30.3)	31 (24.4)	1.87 (1.11–3.13)
Other^2^	2 (0.1)	1 (50.0)	-	2 (0.2)	1 (50.0)	-	0 (0)	-	-
**Race/Ethnicity**									
Asian	608 (39.0)	70 (11.5)	1.17 (0.84–1.62)	415 (36.5)	26 (6.3)	0.68 (0.42–1.07)	193 (46.1)	44 (22.8)	1.93 (1.16–3.24)
White	457 (29.4)	46 (10.1)	0.92 (0.64–1.31)	336 (29.5)	32 (9.5)	1.33 (0.84–2.07)	121 (28.9)	14 (11.6)	0.52 (0.27–0.94)
Latino	286 (18.4)	27 (9.4)	0.86 (0.54–1.30)	232 (20.4)	18 (7.8)	0.96 (0.55–1.61)	54 (12.9)	9 (16.7)	0.92 (0.41–1.90)
Black	29 (1.9)	3 (10.3)	0.97 (0.23–2.80)	25 (2.2)	2 (8.0)	1.00 (0.16–3.46)	4 (1.0)	1 (25.0)	1.56 (0.08–12.39)
Mixed/Other/Not reported	177 (11.4)	19 (10.7)	1.02 (0.60–1.65)	130 (11.4)	13 (10.0)	1.32 (0.68–2.38)	47 (11.2)	6 (12.8)	0.65 (0.24–1.50)
**Comorbidities**									
Any comorbidities	370 (23.8)	41 (11.1)	1.07 (0.73–1.54)	258 (22.7)	18 (7.0)	0.83 (0.47–1.39)	112 (26.7)	23 (20.5)	1.30 (0.74–2.22)
Asthma or COPD	155 (10.0)	16 (10.3)	0.97 (0.54–1.62)	114 (10.0)	9 (7.9)	0.98 (0.45–1.92)	41 (9.8)	7 (17.1)	0.96 (0.38–2.13)
Diabetes mellitus	67 (4.3)	10 (14.9)	1.51 (0.71–2.89)	45 (4.0)	3 (6.7)	0.82 (0.19–2.30)	22 (5.3)	7 (31.8)	2.30 (0.85–5.68)
Hypertension	172 (11.0)	18 (10.5)	0.98 (0.57–1.61)	118 (10.4)	5 (4.2)	0.48 (0.17–1.10)	54 (12.9)	13 (24.1)	1.58 (0.77–3.06)
Smoking or vaping	37 (2.4)	4 (10.8)	1.02 (0.30–2.61)	24 (2.1)	2 (8.3)	1.05 (0.17–3.63)	13 (3.1)	2 (15.4)	0.84 (0.13–3.23)
**COVID-19 exposure outside of work**	58 (3.7)	12 (20.7)	2.29 (1.14–4.29)	28 (2.5)	5 (17.9)	2.59 (0.85–6.47)	30 (7.2)	7 (23.3)	1.46 (0.56–3.39)

^1^ Odds ratios (OR) are unadjusted, comparing the selected group to the entire HCW population

^2^ OR for Other gender omitted due to small sample size (n < 5)

Ab: antibody, HCW: health care worker, CI: confidence interval, rt-PCR: reverse transcriptase polymerase chain reaction, COPD: chronic obstructive pulmonary disease

Potential demographic risk factors identified by bivariate analysis included age, gender, race/ethnicity, and co-morbid conditions, as well as confirmed SARS-CoV-2 exposure outside the hospital (Table 1). No significant effect of age was noted among HCWs overall; however, a non-significant increase in seropositivity was observed for younger HCWs who were not previously tested by rt-PCR. Male gender was associated with increased seropositivity, whereas race/ethnicity and co-morbidities were not. Confirmed COVID-19 exposure outside the hospital was the most significant demographic risk factor for seropositivity.

### Impact of occupational risk factors on SARS-CoV-2 seropositivity

The multivariate model included the non-occupational covariates discussed above, in addition to role and location within the hospital (Table 2). The Hosmer-Lemeshow goodness-of-fit test was non-significant (p = 0.55) and area under the receiver-operating characteristic curve showed moderate discriminant ability (C-statistic = 0.62).

**Table 2:** Associations between HCW occupational factors and SARS-CoV-2 seropositivity of HCW study population and subgroups segregated by prior rt-PCR testing

	All HCWs (n = 1,557)			Not tested by rt-PCR (n = 1,138)			Tested by rt-PCR (n = 419)		
	HCWs, n (%)	COVID-19 AB prevalence, n (%)	Adjusted OR (95% CI)^1^	HCWs, n (%)	COVID-19 AB prevalence, n (%)	Adjusted OR (95% CI)^1^	HCWs, n (%)	COVID-19 AB prevalence, n (%)	Adjusted OR (95% CI)^1^
**Total**	1557 (100)	165 (10.6)		1138 (100)	91 (8.0)		419 (100)	74 (17.7)	
**Role** ^**2**^									
Physician	246 (15.8)	17 (6.9)	0.59 (0.27–1.29)	183 (16.1)	10 (5.5)	0.75 (0.27–2.15)	63 (15.0)	7 (11.1)	0.41 (0.11–1.49)
Nurse	705 (45.3)	90 (12.8)	1.47 (0.81–2.80)	478 (42.0)	40 (8.4)	1.81 (0.81–4.55)	227 (54.2)	50 (22.0)	1.24 (0.49–3.38)
Student	69 (4.4)	5 (7.2)	0.75 (0.22–2.20)	64 (5.6)	5 (7.8)	1.05 (0.28–3.59)	5 (1.2)	0 (0.0)	-
Ancillary clinical staff	88 (5.7)	7 (8.0)	0.91 (0.32–2.36)	55 (4.8)	4 (7.3)	1.48 (0.36–5.32)	33 (7.9)	3 (9.1)	0.46 (0.08–2.19)
Administrative	205 (13.2)	23 (11.2)	1.76 (0.86–3.69)	164 (14.4)	18 (11.0)	2.69 (1.10–7.10)	41 (9.8)	5 (12.2)	0.86 (0.21–3.30)
Food/environmental	46 (3.0)	7 (15.2)	4.85 (1.51–14.85)	37 (3.3)	6 (16.2)	8.28 (2.16–31.48)	9 (2.1)	1 (11.1)	1.52 (0.06–17.75)
Other	199 (12.8)	16 (8.0)	0.70 (0.39–1.18)	157 (13.8)	8 (5.1)	0.54 (0.24–1.09)	42 (10.0)	8 (19.0)	1.19 (0.48–2.65)
**Location** ^**3**^									
COVID ICU	171 (11.0)	26 (15.2)	2.28 (1.29–3.96)	100 (8.8)	10 (10.0)	2.35 (0.97–5.32)	71 (16.9)	16 (22.5)	1.65 (0.71–3.79)
Non-COVID ICU	364 (23.4)	38 (10.4)	0.89 (0.56–1.39)	258 (22.7)	18 (7.0)	0.77 (0.40–1.40)	106 (25.3)	20 (18.9)	1.04 (0.49–2.16)
COVID ward	261 (16.8)	35 (13.4)	1.59 (1.01–2.48)	133 (11.7)	10 (7.5)	1.21 (0.51–2.60)	128 (30.5)	25 (19.5)	1.26 (0.67–2.35)
Non-COVID ward	436 (28.0)	50 (11.5)	1.26 (0.85–1.85)	309 (27.2)	21 (6.8)	0.85 (0.47–1.47)	127 (30.3)	29 (22.8)	2.63 (1.40–4.97)
Labor and delivery	113 (7.3)	4 (3.5)	0.24 (0.06–0.72)	88 (7.7)	4 (4.5)	0.50 (0.12–1.56)	25 (6.0)	0 (0.0)	-
Operating room	196 (12.6)	15 (7.7)	0.99 (0.53–1.78)	173 (15.2)	13 (7.5)	1.13 (0.55–2.17)	23 (5.5)	2 (8.7)	0.76 (0.11–3.43)
Non-operating room procedural	198 (12.7)	16 (8.1)	0.91 (0.49–1.59)	155 (13.6)	14 (9.0)	1.44 (0.72–2.74)	43 (10.3)	2 (4.7)	0.36 (0.05–1.34)
Emergency department	250 (16.1)	20 (8.0)	0.70 (0.40–1.18)	199 (17.5)	13 (6.5)	0.62 (0.31–1.19)	51 (12.2)	7 (13.7)	1.02 (0.36–2.67)
Outpatient clinical unit	188 (12.1)	13 (6.9)	0.70 (0.36–1.27)	143 (12.6)	8 (5.6)	0.72 (0.30–1.53)	45 (10.7)	5 (11.1)	0.63 (0.19–1.73)
Non-clinical unit	249 (16.0)	21 (8.4)	0.70 (0.37–1.24)	201 (17.7)	18 (9.0)	0.76 (0.37–1.51)	48 (11.5)	3 (6.2)	0.47 (0.09–1.76)
**Job-related exposures** ^**4**^									
Cared for COVID patient	599 (38.5)	69 (11.5)	1.10 (0.79–1.54)	396 (34.8)	29 (7.3)	0.85 (0.52–1.34)	203 (48.4)	40 (19.7)	1.13 (0.67–1.92)
3 + days in contact with COVID patient^4^	263 (43.9)	35 (13.3)	1.39 (0.83–2.33)	158 (39.9)	19 (12.0)	2.84 (1.27–6.69)	105 (51.7)	16 (15.2)	0.61 (0.29–1.24)
Participated in aerosol-generating procedure^4^	160 (26.7)	15 (9.4)	0.70 (0.37–1.27)	116 (29.3)	6 (5.2)	0.51 (0.18–1.27)	44 (21.7)	9 (20.5)	1.10 (0.44–2.55)

^1^ Adjusted ORs and 95% CI are adjusted for age, gender, race/ethnicity, known COVID-19 exposure at home, role, location, and whether individual cared for a COVID-19 patient

^2^ Each role is compared to the entire HCW population, e.g., physicians vs. non-physicians

^3^ Individuals may select multiple locations, thus categories are not mutually exclusive. Each aOR corresponds to relative odds of being COVID AB-seropositive for individuals who worked in the specified location versus those who did not

^4^ Days in contact with COVID-19 patient and participated in aerosol-generating procedure only applicable for HCWs who reported “yes” to caring for COVID-19 patients

Ab: antibody, HCW: health care worker, CI: confidence interval, rt-PCR: reverse transcriptase polymerase chain reaction, ICU: intensive care unit

Among roles within the hospital, only HCWs working in food services or environmental services showed significantly increased seropositivity (OR 4.85) as compared to the overall HCW population, and the effect was restricted to those not tested by rt-PCR. Similarly, working in administration was associated with increased seropositivity (OR 2.69) only amongst HCWs not tested by rt-PCR. Among locations in the hospital, working in COVID-19 units was associated with increased seropositivity (OR 2.28 for ICU, 1.59 for ward), whereas working in labor and delivery units was associated with decreased seropositivity (OR 0.24). COVID-19 patient contact and participation in aerosol-generating procedures on these patients were not associated with seropositivity (OR 0.70).

### Correlation of COVID-19 symptoms with seropositivity

A separate multivariable model was constructed that included non-occupational covariates discussed above, in addition to symptoms of COVID-19, but not including occupational covariates (Table 3). Overall, multiple symptoms, specifically fatigue (OR 1.77), myalgias (OR 1.76), fever (OR 1.67), chills (OR 1.79), and anosmia were associated with increased seropositivity, with the strongest association observed for anosmia (OR 5.34). No association between symptoms and seropositivity was observed for HCW who were not previously tested by rt-PCR (i.e. not previously identified by occupational screening).

**Table 3:** Associations between HCW self-reported symptoms and SARS-CoV-2 seropositivity of HCW study population and subgroups segregated by prior rt-PCR testing

	All HCWs (n = 1,557)			Not tested by rt-PCR (n = 1,138)			Tested by rt-PCR (n = 419)		
	HCWs, n (%)	COVID-19 AB prevalence, n (%)	Adjusted OR (95% CI)^1^	HCWs, n (%)	COVID-19 AB prevalence, n (%)	Adjusted OR (95% CI)^1^	HCWs, n (%)	COVID-19 AB prevalence, n (%)	Adjusted OR (95% CI)^1^
**Total**	1557 (100)	165 (10.6)		1138 (100)	91 (8.0)		419 (100)	74 (17.7)	
**Symptoms** ^**2**^									
Sore throat	633 (40.7)	79 (12.5)	1.38 (1.00-1.92)	405 (35.6)	41 (10.1)	1.47 (0.95–2.27)	228 (54.4)	38 (16.7)	0.95 (0.56–1.60)
Fatigue	429 (27.6)	63 (14.7)	1.77 (1.25–2.49)	244 (21.4)	17 (7.0)	0.79 (0.44–1.35)	185 (44.2)	46 (24.9)	2.94 (1.72–5.12)
Muscle aches	361 (23.2)	55 (15.2)	1.76 (1.23–2.50)	198 (17.4)	15 (7.6)	0.89 (0.48–1.56)	163 (38.9)	40 (24.5)	2.20 (1.30–3.74)
New cough	470 (30.2)	54 (11.5)	1.11 (0.78–1.57)	292 (25.7)	18 (6.2)	0.65 (0.36–1.09)	178 (42.5)	36 (20.2)	1.50 (0.89–2.53)
New chills	327 (21.0)	51 (15.6)	1.79 (1.24–2.55)	183 (16.1)	12 (6.6)	0.74 (0.37–1.34)	144 (34.4)	39 (27.1)	2.61 (1.55–4.44)
Fever	318 (20.4)	48 (15.1)	1.67 (1.15–2.39)	177 (15.6)	11 (6.2)	0.67 (0.33–1.24)	141 (33.7)	37 (26.2)	2.38 (1.40–4.04)
Anosmia	95 (6.1)	33 (34.7)	5.34 (3.33–8.45)	40 (3.5)	5 (12.5)	1.67 (0.56–4.08)	55 (13.1)	28 (50.9)	7.67 (4.05–14.76)
Dyspnea	200 (12.8)	27 (13.5)	1.38 (0.87–2.13)	113 (9.9)	8 (7.1)	0.83 (0.36–1.68)	87 (20.8)	19 (21.8)	1.61 (0.86–2.91)

^1^ Adjusted ORs and 95% CI are adjusted for age, gender, race/ethnicity, known COVID-19 exposure at home, role, location, and whether individual cared for COVID-19 patient

^2^ HCWs may report multiple exposures and/or symptoms. Each adjusted OR corresponds to relative odds of being COVID-19 AB seropositive for individuals who reported versus did not report the specified exposure or symptom

Ab: antibody, HCW: health care worker, CI: confidence interval, rt-PCR: reverse transcriptase polymerase chain reaction

## Discussion

**Fig. 1:**
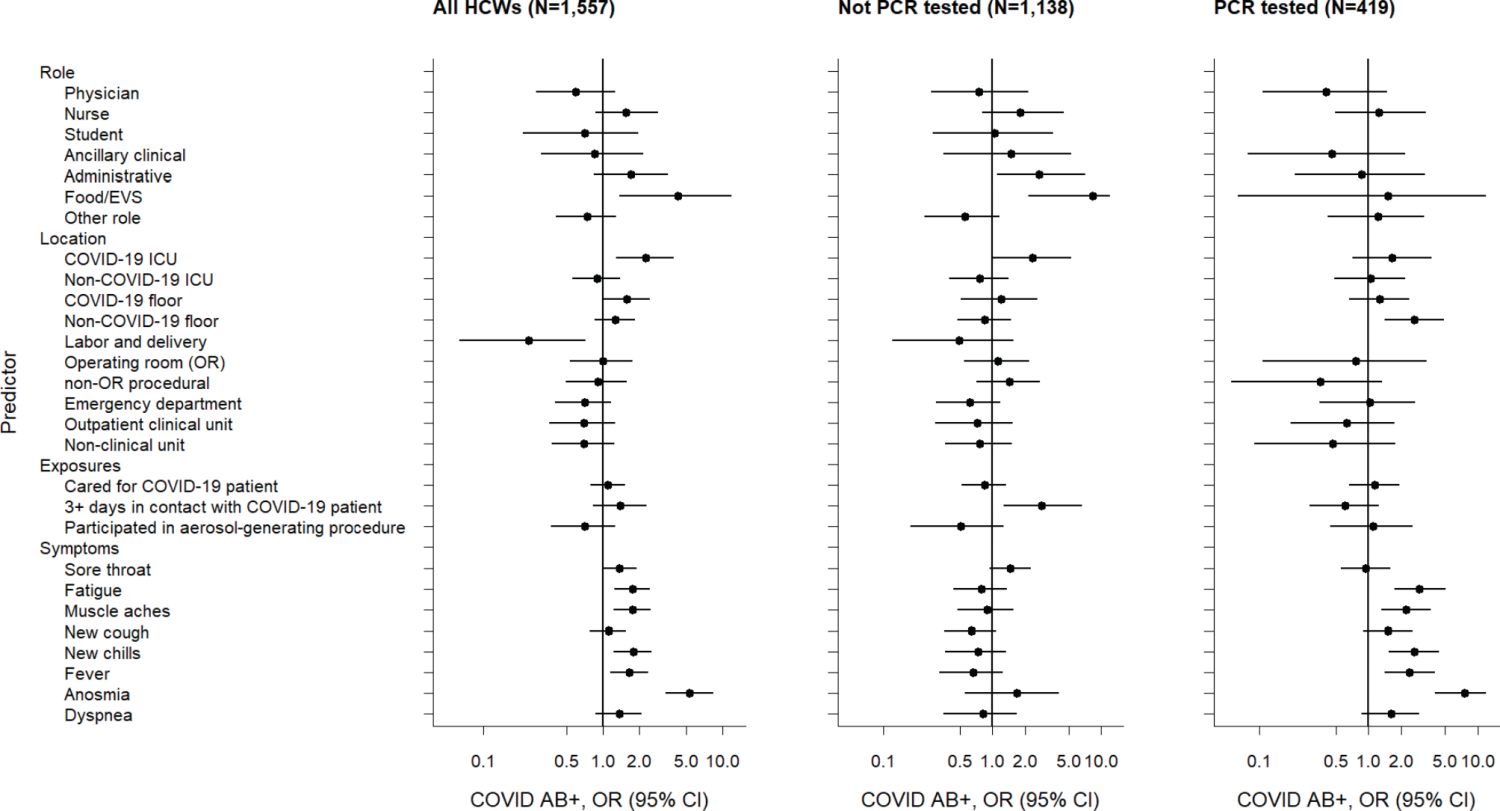
Forest plot of adjusted odds ratios (OR) of hypothesized predictors of COVID-19 seropositivity (AB+) among HCW study population and subgroups segregated by prior rt-PCR testing. ORs are adjusted for sex, age, race/ethnicity, known COVID-19 exposure outside of work, role, location, and COVID-19 patient contact. (EVS, environmental services)

This study provides several insights into the relationships between non-occupational and occupational risk factors and COVID-19 seropositivity among HCWs (Fig. 1). The study hospital was able to maintain infection prevention best practices consistent with guidance from the U.S. Centers for Disease Control and Prevention (CDC), including continuous, ample availability of PPE throughout the pandemic, which is relevant to the question of whether these measures fully prevent in-hospital transmission of SARS-CoV-2. Exposure to COVID-19 outside of work was a greater risk factor for seropositivity than any occupational exposure other than working in food or environmental services. The HCW roles associated with the greatest odds of seropositivity did not involve direct patient care. Nurses, who have the most direct and sustained patient contact, were not at significantly increased odds. The only locations associated with increased seropositivity were the dedicated COVID-19 ICU and floor units. The operating room, an area of great concern due to intubation of multiple patients, was not associated with increased risk. Performing aerosol-generating procedures on known COVID-19 patients was also not significantly associated with seropositivity, which is reassuring given that perceived risk of transmission during these procedures can delay patient care.

**Fig. 2:**
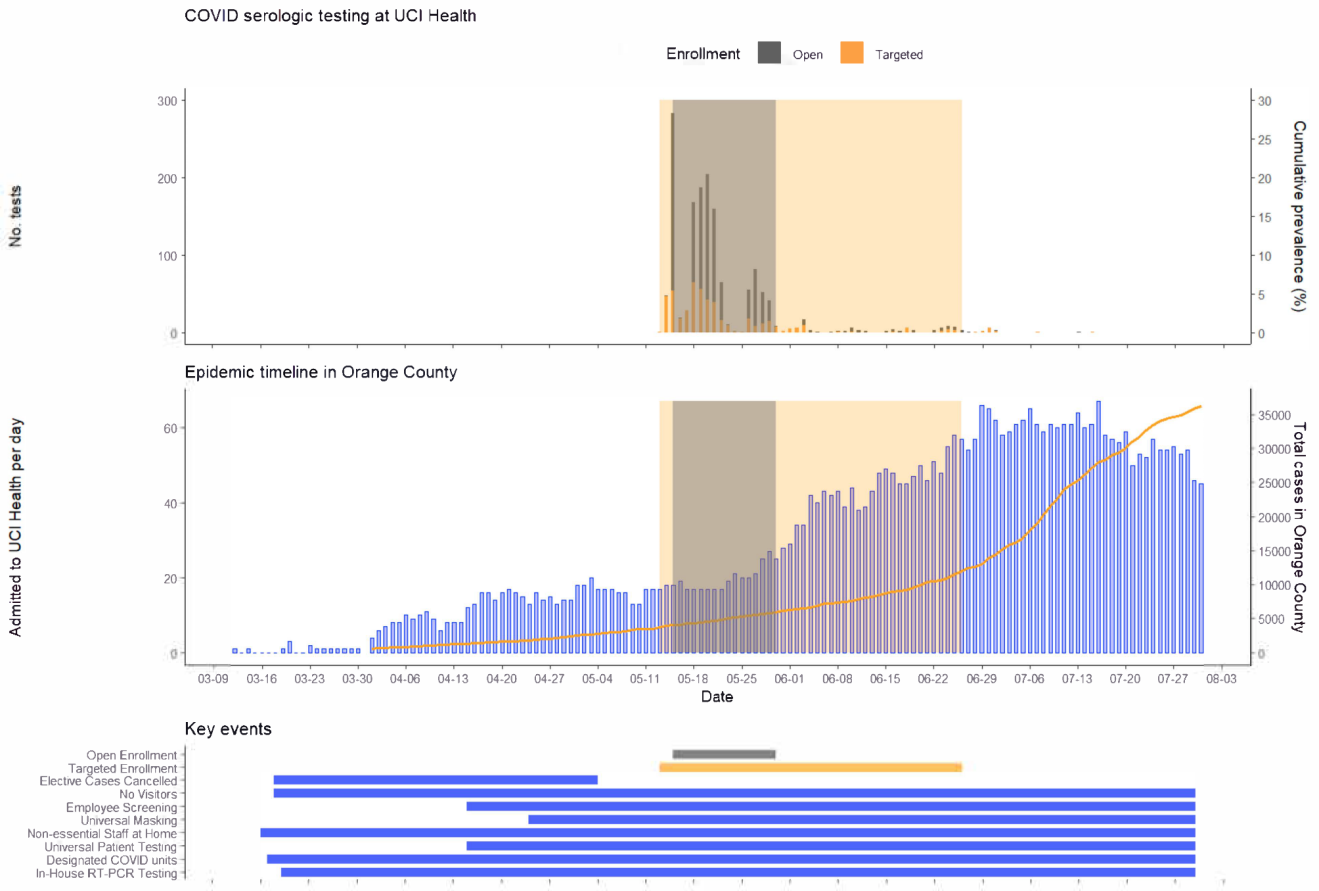
Epidemiologic context of HCW study with respect to community prevalence and hospital burden of COVID-19. This study was performed between May 15th to June 30th, 2020 (top graph), a period in which total cases in Orange County were on a rise (bottom graph). Key events for the University Hospital system are outlined in the bottom

Stratification of HCWs based on whether or not they were tested previously by rt-PCR yielded several additional insights into the strengths and weaknesses of universal symptom screening. The study hospital was conducting universal symptom screening of all HCWs during the study period, and HCW who screened positive were captured in the subgroup tested by rt-PCR; those HCW who did not screen positive for symptoms and were not tested by rt-PCR but were found to be seropositive likely reflect asymptomatic infections or lack of reporting of symptoms. Although the hospital’s mandatory occupational health screening was only implemented one month prior to this study, relatively few COVID-19 infections would have occurred prior to this screening based on the local prevalence of COVID-19 (Fig. 2). The association between COVID-19 symptoms and seropositivity was restricted to HCW tested previously by rt-PCR, indicating that universal screening was effective in identifying symptomatic infections among the HCW in the study population. Younger HCWs who were COVID-19-seropositive were more likely to be missed by occupational screening, which is consistent with the increased prevalence of minimally symptomatic infection among younger individuals [33]. Decreased seropositivity among HCW in labor and delivery units may be due to increased vigilance amongst HCW who care for pregnant patients or low disease prevalence among these patients. Exposure to COVID-19 outside of work was associated with increased odds of seropositivity among HCWs not previously tested by rt-PCR, indicating that these exposures were not being universally reported during screening as they should have prompted rt-PCR testing.

While no data were available for SARS-CoV-2 seropositivity in the surrounding community at the time that the study was performed, the prevalence of COVID-19 in Orange County was 0.2% at that time (based on case reporting to the Orange County Health Department) and increased subsequently (Fig. 2). The overall seropositivity rate was 10.8% in this study, but the 8.0% seropositivity amongst HCW not previously identified by screening, which matches the seropositivity in the open enrollment cohort, is most appropriate for comparison to community prevalence to avoid the enrichment effect of the targeted enrollment cohort. This estimated seropositivity is 40-fold higher than community prevalence based on public health case reporting confirmed by rt-PCR testing. This large discrepancy is likely explained in part by the waning of PCR positivity over time, as viral shedding is short-term whereas seropositivity is relatively sustained. Whereas more recent seroprevalence studies show a lower increase compared to case counts, our result is most comparable to early seroprevalence studies prior to significant local outbreaks of COVID-19 that have found larger disparities between seropositivity rates and case counts [6–10, 16]. Subsequently, a community study sampled 2,979 random participants in Orange County from July 10 through August 16, 2020 and found a seropositivity rate of 11.5% (95% CI: 10.5–12.4%) using an updated version of the CoVAM with a more stringent threshold for seropositivity [34]. The seroprevalence estimates imply that HCWs have SARS-CoV-2 seropositivity similar to the surrounding community (although seropositivity in both studies is much higher than prevalence based on public health case reporting confirmed by rt-PCR testing), with the caveat that the two studies were performed during different time periods.

The findings of this study are largely consistent with recently published studies of SARS-CoV-2 seropositivity among HCWs [22–25]. In particular, the lack of association of either race/ethnicity or co-morbidities with seropositivity in our study is consistent with these prior HCW studies and differs from a prior community study that did observe such associations [35]. This study provides additional insight compared to prior studies by examining specific roles in the hospital and controlling for multiple likely sources of confounding. For example, nurses had significantly elevated seropositivity in bivariate analyses (unadjusted OR [CI] = 1.52 [1.10–2.10]) but this finding did not persist after adjusting for work location; in contrast, null associations between seropositivity and roles without direct patient care became significant and positive after adjusting for location (unadjusted OR [CI] for administrative = 1.08 [0.66, 1.69]; food/environmental = 1.54 [0.62–3.29]).

Strengths of this study include the validated test performance of the CoVAM, which compares favorably to currently available single-antigen assays; the large sample size with inclusion of 34.4% of HCW at the hospital; and the use of multivariable analysis to control for confounding. The weaknesses of this study include the non-random enrollment methodology as the targeted enrollment cohort was invited from groups expected to have higher seroprevalence and the open enrollment cohort was subject to self-selection, which both could lead to sampling bias. Also, different blood sampling methodology was used in the open and targeted enrollment cohorts due to institutional interest in banking specimens from the latter group. The subgroup analysis based on prior rt-PCR testing was used to control for the heterogeneous sampling, as prior testing was the primary driver of increased prevalence in the targeted enrollment cohort. When the study population is stratified based on method of recruitment (Supplementary Tables 1–3 and Supplementary Fig. 1 ), the results are largely similar to stratification based on rt-PCR testing (Tables 1, 2 and 3; Fig. 1).

## Conclusion

The results of this study have several implications for the local and global responses to the COVID-19 pandemic. The finding of a significantly increased SARS-CoV-2 prevalence by serology as compared with rt-PCR provides evidence that the reported counts of confirmed COVID-19 cases are significant underestimates. The observations that HCWs who are younger, work in non-patient care roles, or have COVID-19 exposure outside of work are more likely to have COVID-19 seropositivity without prior testing indicates that screening and vaccination efforts targeting these groups can be particularly effective. While we do not observe an association of aerosol-generating procedures with SARS-CoV-2 seropositivity in the context of adequate availability and presumably appropriate use of PPE, we do observe increased seropositivity in COVID-19 units, but this may potentially be related to geographical factors other than patient care given that caring for COVID-19 patients was not a significant risk factor. Further studies are needed to confirm these observations.

Of note, this study was performed prior to the availability of vaccines against SARS-CoV-2, which is now required for workers in most healthcare facilities. While these early pandemic observations are therefore less relevant to the current epidemiology of the COVID-19 pandemic in healthcare facilities, they can be used to inform the hospital epidemiology response to future epidemics of viral respiratory infections.

## Electronic supplementary material

Below is the link to the electronic supplementary material.

Supplementary Material 1

Supplementary Material 2

## Data Availability

The dataset generated by testing specimens on the coronavirus antigen microarray and the analysis code applied to this dataset is available upon request. The associated clinical data with removal of all identifying information is also available upon request, please contact Dr. Saahir Khan at Saahir.khan@med.usc.edu for data requests.
