# Supplementary material for: Risk factors for SARS-CoV-2 seropositivity in a health care worker population during the early pandemic
Source: BMC Infect Dis. 2023 May 16;23:330. doi: 10.1186/s12879-023-08284-y (PMC10186297; doi:10.1186/s12879-023-08284-y)
Supplement: Supplementary file 1 — Supplementary Material 1 [file 12879_2023_8284_MOESM1_ESM.docx]

**Supplementary Information**

**Supplementary Tables**

**Supplementary Table 1. Association between demographic and health-related characteristics and SARS-CoV-2 seropositivity (AB prevalence) of HCW study population and subgroups segregated by enrollment group. (COPD, chronic obstructive pulmonary disease)**

|  | | **All HCWs (n=1,557)** | | | **Open enrollment (n=1,044)** | | | | **Targeted enrollment (n=513)** | | |
| --- | --- | --- | --- | --- | --- | --- | --- | --- | --- | --- | --- |
|  | **HCWs,**  **n (%)** | **COVID-19 AB prevalence,**  **n (%)** | **OR (95% CI)^1^** | **HCWs,**  **n (%)** | | **COVID-19 AB prevalence,**  **n (%)** | **OR (95% CI)^1^** | **HCWs,**  **n (%)** | | **COVID-19 AB prevalence,**  **n (%)** | **OR (95% CI)^1^** |
| **Total** | 1557 (100.0) | 165 (10.6) |  | 1044 (100.0) | | 83 (8.0) |  | 513 (100.0) | | 82 (16.0) |  |
| **Age quartiles (y)** |  |  |  |  | |  |  |  | |  |  |
| 18-31 | 418 (26.8) | 49 (11.7) | 1.17 (0.82-1.66) | 268 (25.7) | | 23 (8.6) | 1.12 (0.67-1.83) | 150 (29.2) | | 26 (17.3) | 1.15 (0.68-1.90) |
| 32-38 | 382 (24.5) | 41 (10.7) | 1.02 (0.69-1.47) | 244 (23.4) | | 24 (9.8) | 1.37 (0.82-2.23) | 138 (26.9) | | 17 (12.3) | 0.67 (0.37-1.17) |
| 39-43 | 377 (24.2) | 35 (9.3) | 0.83 (0.55-1.21) | 258 (24.7) | | 15 (5.8) | 0.65 (0.35-1.13) | 119 (23.2) | | 20 (16.8) | 1.08 (0.61-1.85) |
| 49-73 | 380 (24.4) | 40 (10.5) | 0.99 (0.67-1.43) | 274 (26.2) | | 21 (7.7) | 0.95 (0.55-1.56) | 106 (20.7) | | 19 (17.9) | 1.19 (0.66-2.06) |
| **Gender** |  |  |  |  | |  |  |  | |  |  |
| Female | 1073 (68.9) | 100 (9.3) | 0.66 (0.48-0.93) | 696 (66.7) | | 47 (6.8) | 0.63 (0.40-0.99) | 377 (73.5) | | 53 (14.1) | 0.60 (0.37-1.01) |
| Male | 482 (31.0) | 64 (13.3) | 1.48 (1.05-2.06) | 347 (33.2) | | 35 (10.1) | 1.52 (0.96-2.39) | 135 (26.3) | | 29 (21.5) | 1.68 (1.01-2.76) |
| Other^2^ | 2 (0.1) | 1 (50.0) | - | 1 (0.1) | | 1 (100) | - | 1 (0.1) | | 0 | - |
| **Race/ethnicity** |  |  |  |  | |  |  |  | |  |  |
| Asian | 608 (39.0) | 70 (11.5) | 1.17 (0.84-1.62) | 357 (34.2) | | 23 (6.4) | 0.72 (0.43-1.17) | 251 (48.9) | | 47 (18.7) | 1.49 (0.93-2.42) |
| White | 457 (29.4) | 46 (10.1) | 0.92 (0.64-1.31) | 332 (31.8) | | 30 (9.0) | 1.24 (0.77-1.96) | 125 (24.4) | | 16 (12.8) | 0.72 (0.39-1.26) |
| Latino | 286 (18.4) | 27 (9.4) | 0.86 (0.54-1.30) | 228 (21.8) | | 17 (7.5) | 0.92 (0.51-1.56) | 58 (11.3) | | 10 (17.2) | 1.11 (0.51-2.21) |
| Black | 29 (1.9) | 3 (10.3) | 0.97 (0.23-2.80) | 23 (2.2) | | 2 (8.7) | 1.11 (0.17-3.86) | 6 (1.2) | | 1 (16.7) | 1.05 (0.05-6.63) |
| Mixed/Other/Not reported | 177 (11.4) | 19 (10.7) | 1.02 (0.60-1.65) | 104 (10.0) | | 11 (10.6) | 1.43 (0.69-2.68) | 73 (14.2) | | 8 (11.0) | 0.61 (0.26-1.25) |
| **Comorbidities** |  |  |  |  | |  |  |  | |  |  |
| Any comorbidities | 370 (23.8) | 41 (11.1) | 1.07 (0.73-1.54) | 243 (23.3) | | 20 (8.2) | 1.05 (0.61-1.75) | 127 (24.8) | | 21 (16.5) | 1.06 (0.60-1.79) |
| Asthma or COPD | 155 (10.0) | 16 (10.3) | 0.97 (0.54-1.62) | 102 (9.8) | | 9 (8.8) | 1.14 (0.52-2.23) | 53 (10.3) | | 7 (13.2) | 0.78 (0.31-1.69) |
| Diabetes mellitus | 67 (4.3) | 10 (14.9) | 1.51 (0.71-2.89) | 41 (3.9) | | 4 (9.8) | 1.26 (0.37-3.25) | 26 (5.1) | | 6 (23.1) | 1.62 (0.58-3.95) |
| Hypertension | 172 (11.0) | 18 (10.5) | 0.98 (0.57-1.61) | 115 (11.0) | | 7 (6.1) | 0.73 (0.30-1.51) | 57 (11.1) | | 11 (19.3) | 1.30 (0.61-2.54) |
| Smoking or vaping | 37 (2.4) | 4 (10.8) | 1.02 (0.30-2.61) | 23 (2.2) | | 1 (4.3) | 0.52 (0.03-2.53) | 14 (2.7) | | 3 (21.4) | 1.45 (0.32-4.77) |
| **COVID-19 exposure outside of work** | 58 (3.7) | 12 (20.7) | 2.29 (1.14-4.29) | 33 (3.2) | | 6 (18.2) | 2.70 (0.98-6.32) | 25 (4.9) | | 6 (24.0) | 1.71 (0.61-4.20) |

^1^ Odds ratios (OR) are unadjusted, comparing the selected group to the entire HCW population.

^2^ OR for Other gender omitted due to small sample size.

**Supplementary Table 2. Associations between HCW occupational factors and SARS-CoV-2 seropositivity (AB prevalence) of HCW study population and subgroups segregated by enrollment group.**

|  | **All HCWs (n=1,557)** | | | **Open enrollment/fingerstick (n=1044)** | | | **Targeted enrollment/EIP (n=513)** | | |
| --- | --- | --- | --- | --- | --- | --- | --- | --- | --- |
|  | **HCWs,**  **n (%)** | **COVID-19 AB prevalence, n (%)** | **Adjusted OR (95% CI)^1^** | **HCWs,**  **n (%)** | **COVID-19 AB prevalence, n (%)** | **Adjusted OR (95% CI)^1^** | **HCWs,**  **n (%)** | **COVID-19 AB prevalence, n (%)** | **Adjusted OR (95% CI)^1^** |
| **Total** | 1557 (100.0) | 165 (10.6) |  | 1044 (100.0) | 83 (8.0) |  | 513 (100.0) | 82 (16.0) |  |
| **Role^2^** |  |  |  |  |  |  |  |  |  |
| Physician | 246 (15.8) | 17 (6.9) | 0.59 (0.28-1.27) | 170 (16.3) | 11 (6.5) | 0.74 (0.28-1.96) | 76 (14.8) | 6 (7.9) | 0.26 (0.06-1.04) |
| Nurse | 705 (45.3) | 90 (12.8) | 1.55 (0.87-2.91) | 370 (35.4) | 32 (8.6) | 1.44 (0.66-3.39) | 335 (65.3) | 58 (17.3) | 1.22 (0.48-3.49) |
| Student | 69 (4.4) | 5 (7.2) | 0.70 (0.21-1.98) | 69 (6.6) | 5 (7.2) | 0.77 (0.21-2.52) | 0 (0) | - | - |
| Ancillary clinical staff | 88 (5.7) | 7 (8.0) | 0.85 (0.30-2.18) | 60 (5.7) | 3 (5.0) | 0.84 (0.17-3.20) | 28 (5.5) | 4 (14.3) | 0.90 (0.19-3.75) |
| Administrative | 205 (13.2) | 23 (11.2) | 1.71 (0.84-3.52) | 171 (16.4) | 15 (8.8) | 1.71 (0.71-4.25) | 34 (6.6) | 8 (23.5) | 1.57 (0.44-5.66) |
| Food / environmental | 46 (3.0) | 7 (15.2) | 4.26 (1.37-12.61) | 46 (4.4) | 7 (15.2) | 6.30 (1.84-21.39) | 0 (0) | - | - |
| Other | 199 (12.8) | 16 (8.0) | 0.70 (0.39-1.18) | 158 (15.1) | 10 (6.3) | 0.72 (0.34-1.38) | 41 (8.0) | 6 (14.6) | 0.93 (0.34-2.18) |
| **Location^3^** |  |  |  |  |  |  |  |  |  |
| COVID-19 ICU | 171 (11.0) | 26 (15.2) | 2.28 (1.29-3.96) | 98 (9.4) | 9 (9.2) | 2.49 (0.89-6.39) | 73 (14.2) | 17 (23.3) | 2.07 (0.95-4.48) |
| Non-COVID-19 ICU | 364 (23.4) | 38 (10.4) | 0.89 (0.56-1.39) | 254 (24.3) | 17 (6.7) | 0.74 (0.36-1.40) | 110 (21.4) | 21 (19.1) | 1.25 (0.62-2.47) |
| COVID-19 floor | 261 (16.8) | 35 (13.4) | 1.59 (1.01-2.48) | 131 (12.5) | 10 (7.6) | 1.25 (0.49-2.94) | 130 (25.3) | 25 (19.2) | 1.68 (0.91-3.08) |
| Non-COVID-19 floor | 436 (28.0) | 50 (11.5) | 1.26 (0.85-1.85) | 282 (27.0) | 24 (8.5) | 1.22 (0.68-2.14) | 154 (30.0) | 26 (16.9) | 1.67 (0.91-3.03) |
| Labor and delivery | 113 (7.3) | 4 (3.5) | 0.24 (0.06-0.72) | 102 (9.8) | 4 (3.9) | 0.35 (0.08-1.13) | 11 (2.1) | 0 | - |
| Operating room | 196 (12.6) | 15 (7.7) | 0.99 (0.53-1.78) | 183 (17.5) | 13 (7.1) | 1.00 (0.48-1.93) | 13 (2.5) | 2 (15.4) | 3.34 (0.43-18.11) |
| non-operating room procedural | 198 (12.7) | 16 (8.1) | 0.91 (0.49-1.59) | 180 (17.2) | 14 (7.8) | 1.10 (0.54-2.09) | 18 (3.5) | 2 (11.1) | 0.75 (0.10-3.25) |
| Emergency department | 250 (16.1) | 20 (8.0) | 0.70 (0.40-1.18) | 143 (13.7) | 9 (6.3) | 0.68 (0.28-1.47) | 107 (20.9) | 11 (10.3) | 0.76 (0.33-1.68) |
| Outpatient clinical unit | 188 (12.1) | 13 (6.9) | 0.70 (0.36-1.27) | 162 (15.5) | 10 (6.2) | 0.82 (0.37-1.66) | 26 (5.1) | 3 (11.5) | 0.87 (0.19-2.92) |
| Non-clinical unit | 249 (16.0) | 21 (8.4) | 0.70 (0.37-1.24) | 223 (21.4) | 16 (7.2) | 0.60 (0.29-1.19) | 26 (5.1) | 5 (19.2) | 2.08 (0.55-7.07) |
| **Job-related exposures^2^** |  |  |  |  |  |  |  |  |  |
| Cared for COVID-19 patient | 599 (38.5) | 69 (11.5) | 1.10 (0.79-1.54) | 305 (29.2) | 21 (6.9) | 0.74 (0.43-1.24) | 294 (57.3) | 48 (16.3) | 1.02 (0.63-1.69) |
| 3+ days in contact with COVID-19 patient^4^ | 263 (43.9) | 35 (13.3) | 1.39 (0.83-2.33) | 121 (39.7) | 7 (5.8) | 0.70 (0.25-1.78) | 142 (48.3) | 28 (19.7) | 1.74 (0.92-3.36) |
| Participated in aerosol-generating procedure^4^ | 160 (26.7) | 15 (9.4) | 0.70 (0.37-1.27) | 84 (27.5) | 5 (6.0) | 0.70 (0.22-1.96) | 76 (25.9) | 10 (13.2) | 0.69 (0.30-1.46) |

^1^ Adjusted ORs and 95% CI are adjusted for age, gender, race/ethnicity, known COVID exposure at home, role, location, and whether individual cared for COVID patient.

^2^ Each role is compared to the entire HCW population, e.g., physicians vs. non-physicians.

^3^ Individuals may select multiple locations, thus categories are not mutually exclusive. Each aOR corresponds to relative odds of being COVID AB-seropositive for individuals who worked in the specified location versus those who did not.

^4^ Days in contact with COVID patient and participated in aerosol-generating procedure only applicable for HCWs who reported “yes” to caring for COVID patients.

**Supplementary Table 3. Association between HCW self-reported symptoms and SARS-CoV-2 seropositivity (AB prevalence) of HCW study population and subgroups segregated by enrollment group.**

|  | **All HCWs (n=1,557)** | | | **Open enrollment (n=1,044)** | | | **Targeted enrollment (n=513)** | | |
| --- | --- | --- | --- | --- | --- | --- | --- | --- | --- |
|  | **HCWs, n (%)** | **COVID-19 AB prevalence, n (%)** | **Adjusted OR (95% CI)^1^** | **HCWs, n (%)** | **COVID-19 AB prevalence, n (%)** | **Adjusted OR (95% CI)^1^** | **HCWs, n (%)** | **COVID-19 AB prevalence, n (%)** | **Adjusted OR**  **(95% CI)^1^** |
| **Total** | 1557 (100.0) | 165 (10.6) |  | 1044 (100.0) | 83 (8.0) |  | 513 (100.0) | 82 (16.0) |  |
| **Symptoms^2^** |  |  |  |  |  |  |  |  |  |
| Sore throat | 633 (40.7) | 79 (12.5) | 1.38 (1.00-1.92) | 415 (39.8) | 35 (8.4) | 1.07 (0.67-1.70) | 218 (42.5) | 44 (20.2) | 1.72 (1.06-2.79) |
| Fatigue | 429 (27.6) | 63 (14.7) | 1.77 (1.25-2.49) | 259 (24.8) | 20 (7.7) | 0.91 (0.52-1.54) | 170 (33.1) | 43 (25.3) | 2.75 (1.68-4.52) |
| Muscle aches | 361 (23.2) | 55 (15.2) | 1.76 (1.23-2.50) | 216 (20.7) | 15 (6.9) | 0.77 (0.41-1.35) | 145 (28.3) | 40 (27.6) | 2.95 (1.79-4.88) |
| New cough | 470 (30.2) | 54 (11.5) | 1.11 (0.78-1.57) | 305 (29.2) | 22 (7.2) | 0.81 (0.47-1.33) | 165 (32.2) | 32 (19.4) | 1.41 (0.85-2.31) |
| New chills | 327 (21.0) | 51 (15.6) | 1.79 (1.24-2.55) | 203 (19.4) | 17 (8.4) | 0.99 (0.54-1.70) | 124 (24.2) | 34 (27.4) | 2.59 (1.55-4.29) |
| Fever | 318 (20.4) | 48 (15.1) | 1.67 (1.15-2.39) | 191 (18.3) | 15 (7.9) | 0.90 (0.48-1.59) | 127 (24.8) | 33 (26.0) | 2.34 (1.40-3.86) |
| Loss of smell | 95 (6.1) | 33 (34.7) | 5.34 (3.33-8.45) | 51 (4.9) | 9 (17.6) | 2.47 (1.07-5.12) | 44 (8.6) | 24 (54.5) | 8.50 (4.37-16.75) |
| Dyspnea | 200 (12.8) | 27 (13.5) | 1.38 (0.87-2.13) | 131 (12.5) | 12 (9.2) | 1.11 (0.54-2.08) | 69 (13.5) | 15 (21.7) | 1.49 (0.77-2.77) |

^1^ Adjusted ORs and 95% CI are adjusted for age, gender, race/ethnicity, known COVID-19 exposure at home, role, location and whether an individual cared for a COVID-19 patient.

^2^ HCWs may have reported multiple exposures or symptoms. Each adjusted OR corresponds to relative odds of being COVID-19 AB-seropositive for individuals who reported versus did not report the specified exposure or symptom.

**Supplementary Table 4. Demographic and health-related characteristics and SARS-CoV-2 seropositivity of HCW subgroups without prior rt-PCR testing and with prior positive and negative rt-PCR testing.**

|  | **Not tested for PCR** | | **PCR Positive** | | **PCR Negative** | |
| --- | --- | --- | --- | --- | --- | --- |
|  | **HCWs, n (%)** | **COVID AB+, n (%)** | **HCWs, n (%)** | **COVID AB+, n (%)** | **HCWs, n (%)** | **COVID AB+, n (%)** |
| **Total** | 1138 (100.0) | 91 (8.0) | 38 (100.0) | 36 (94.7) | 322 (100.0) | 30 (9.3) |
| **Age quartiles (y)** |  |  |  |  |  |  |
| 18-31 | 311 (27.3) | 33 (10.6) | 7 (18.4) | 6 (85.7) | 86 (26.7) | 7 (8.1) |
| 32-38 | 257 (22.6) | 22 (8.6) | 9 (23.7) | 8 (88.9) | 98 (30.4) | 10 (10.2) |
| 39-43 | 275 (24.2) | 15 (5.5) | 11 (28.9) | 11 (100.0) | 74 (23.0) | 7 (9.5) |
| 49-73 | 295 (25.9) | 21 (7.1) | 11 (28.9) | 11 (100.0) | 64 (19.9) | 6 (9.4) |
| **Gender** |  |  |  |  |  |  |
| Female | 781 (68.6) | 57 (7.3) | 22 (57.9) | 21 (95.5) | 228 (70.8) | 18 (7.9) |
| Male | 355 (31.2) | 33 (9.3) | 16 (42.1) | 15 (93.8) | 94 (29.2) | 12 (12.8) |
| **Race/ethnicity** |  |  |  |  |  |  |
| Asian | 415 (36.5) | 26 (6.3) | 23 (60.5) | 22 (95.7) | 151 (46.9) | 19 (12.6) |
| White | 336 (29.5) | 32 (9.5) | 6 (15.8) | 6 (100.0) | 92 (28.6) | 6 (6.5) |
| Latino | 232 (20.4) | 18 (7.8) | 7 (18.4) | 7 (100.0) | 45 (14.0) | 1 (2.2) |
| Black | 25 (2.2) | 2 (8.0) | NA | NA | 3 (0.9) | 1 (33.3) |
| Mixed/Other/Not reported | 130 (11.4) | 13 (10.0) | 2 (5.3) | 1 (50.0) | 31 (9.6) | 3 (9.7) |
| **Comorbidities** |  |  |  |  |  |  |
| Any comorbidities | 258 (22.7) | 18 (7.0) | 14 (36.8) | 14 (100.0) | 80 (24.8) | 7 (8.8) |
| Asthma or COPD | 114 (10.0) | 9 (7.9) | 3 (7.9) | 3 (100.0) | 31 (9.6) | 2 (6.5) |
| Diabetes mellitus | 45 (4.0) | 3 (6.7) | 4 (10.5) | 4 (100.0) | 14 (4.3) | 3 (21.4) |
| Hypertension | 118 (10.4) | 5 (4.2) | 9 (23.7) | 9 (100.0) | 39 (12.1) | 4 (10.3) |
| Smoking or vaping | 24 (2.1) | 2 (8.3) | 1 (2.6) | 1 (100.0) | 10 (3.1) | 1 (10.0) |
| **COVID-19 exposure outside of work** | 28 (2.5) | 5 (17.9) | 5 (13.2) | 5 (100.0) | 24 (7.5) | 2 (8.3) |

**Supplementary Figures**


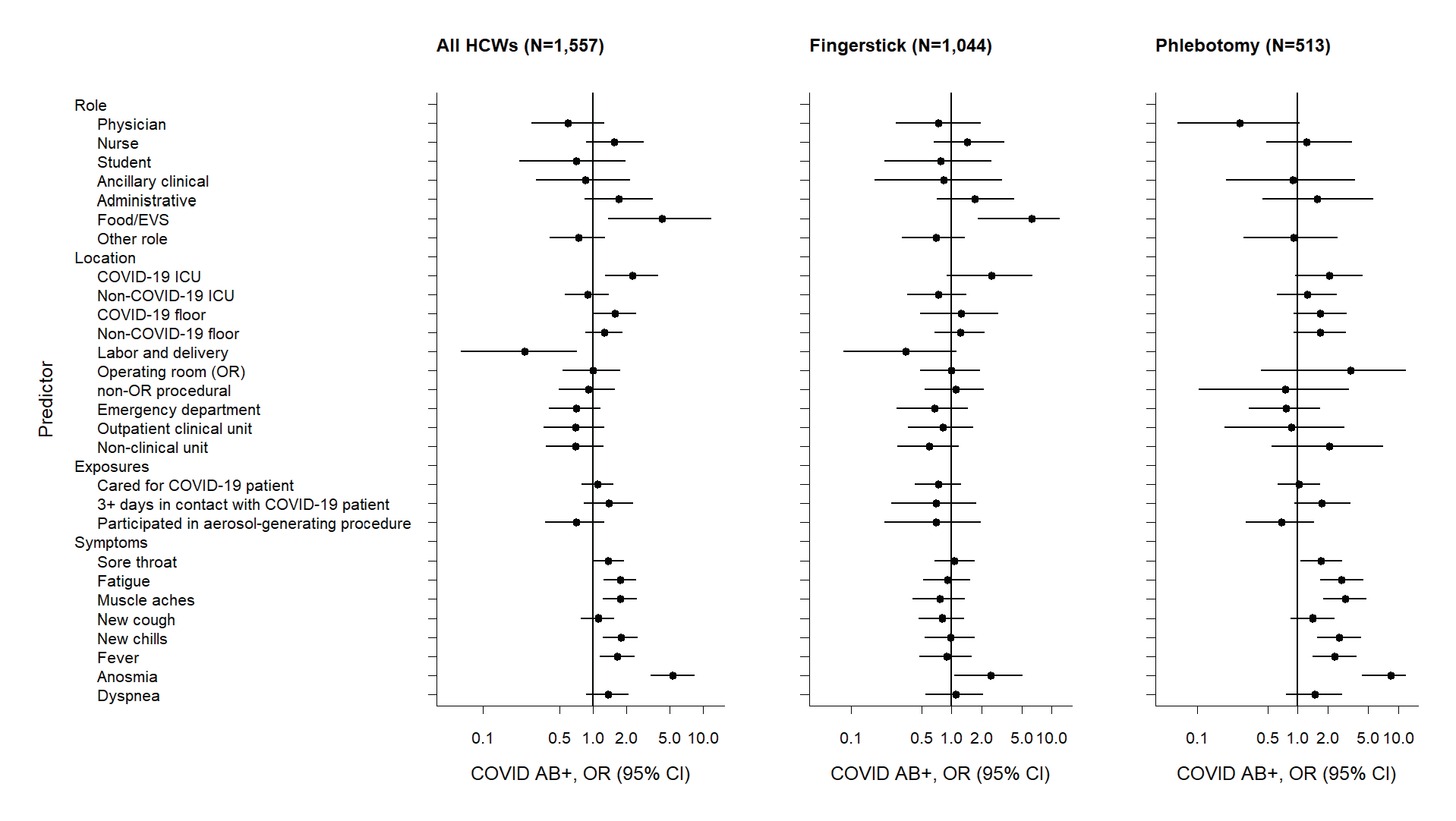


**Supplementary Figure 1.** Forest plot of adjusted odds ratios (OR) of hypothesized predictors of COVID-19 seropositivity (AB+) among HCW study population and subgroups segregated by enrollment group and sample collection method (Fingerstick = open enrollment cohort; Phlebotomy = targeted enrollment cohort). ORs are adjusted for sex, age, race/ethnicity, known COVID-19 exposure outside of work, role, location, and COVID-19 patient contact. (EVS, environmental services)
