# Supplementary material for: Risk factors for SARS-CoV-2 seropositivity in a health care worker population during the early pandemic
Source: BMC Infect Dis. 2023 May 16;23:330. doi: 10.1186/s12879-023-08284-y (PMC10186297; doi:10.1186/s12879-023-08284-y)
Supplement: Supplementary file 2 — Supplementary Material 2 [file 12879_2023_8284_MOESM2_ESM.pdf]

Thanks for your participation! Please complete the survey below.

What is your first name (as listed in HR)?

____

What is your last name (as listed in HR)?

____

What is your role/title at UCI Health?

- ◯ Administrative Staff
- ◯ Admitting & Registration/Billing
- ◯ Case Management/Social Worker
- ◯ Dietary / Food Services
- ◯ EIP
- ◯ Emergency Medical Technician
- ◯ EVS
- ◯ IT Finance
- ◯ Nurse
- ◯ Nurse Aide
- ◯ Nutritionist
- ◯ Occupational Therapist
- ◯ Pastoral Care
- ◯ Patient Experience
- ◯ Patient Safety & Quality
- ◯ Pharmacist
- ◯ Phlebotomist
- ◯ Physical Therapist
- ◯ Physician
- ◯ Researcher
- ◯ Respiratory Therapist
- ◯ Security
- ◯ Student
- ◯ Telemetry, Radiology or Laboratory Technician
- ◯ Unit Secretary
- ◯ Other

What is your title?

- ◯ Faculty
- ◯ Resident
- ◯ Fellow

Are you an ICU Nurse or non-ICU Nurse?

- ◯ ICU Nurse
- ◯ non-ICU Nurse

Please provide your email address: __________________________________

Please provide your badge number (5 digit serial number on ID card): __________________________________

How long have you worked at UCI Health (estimated in years)? __________________________________

Please provide the unit, area or floor where you perform most of your work (select all that apply):

- ☐ Acute Rehabilitation Unit
- ☐ Administration
- ☐ Adolescent Partial Hospitalization Program
- ☐ Adolescent Psychiatric Unit
- ☐ Blood bank
- ☐ Burn Intensive Care Unit (BICU)
- ☐ Cardiac Catheterization
- ☐ Cardiac Intensive Care Unit (CCU)
- ☐ Cafeteria
- ☐ Center for Digestive Diseases
- ☐ Center for Perioperative Care (CPC)
- ☐ Central Sterile Processing Unit
- ☐ Clinical Laboratory
- ☐ Dietary / Food Services
- ☐ DH 32 (Orthopedics)
- ☐ DH 46/48 (Mother/Baby Unit)
- ☐ DH 56 (Neuroscience Step Down Unit)
- ☐ DH 58 (Medical-Surgical: Neuro/Trauma)
- ☐ DH 66/68 (Surgical Step Down Unit/Telemetry)
- ☐ DH 76 (Oncology)
- ☐ DH 78 (Telemetry)
- ☐ Emergency Department (ED)
- ☐ EVS
- ☐ Infusion Center
- ☐ Interventional Radiology
- ☐ Medical Intensive Care Unit (MICU)
- ☐ Medical Psychiatric Unit
- ☐ Neonatal Intensive Care Unit (NICU)
- ☐ Neuroscience Intensive Care Unit (NSCU)
- ☐ Operating Room
- ☐ Outpatient Surgical Services (OSS)
- ☐ Post-Anesthesia Care Unit (PACU)
- ☐ Pre- and Post-Operative Care Unit (PPCU)
- ☐ Radiology (XR, CT, MRI)
- ☐ Surgical Intensive Unit (SICU)
- ☐ 2 Tower (Antepartum)
- ☐ 2 Tower (Labor and Delivery)
- ☐ 3 Tower (Telemetry)
- ☐ 4 Tower (Medical)
- ☐ 5 Tower (Telemetry)

What is your age?

____

Gender

- ◯ Female
- ◯ Male
- ◯ Other

Race/Ethnicity

- ☐ American Indian/Alaska Native
- ☐ Asian
- ☐ Black or African American
- ☐ Latino or Hispanic
- ☐ Native Hawaiian or Other Pacific Islander
- ☐ White
- ☐ More Than One Race
- ☐ Unknown / Not Reported

Height

◯ 4'0"
◯ 4'1"
◯ 4'2"
◯ 4'3"
◯ 4'4"
◯ 4'5"
◯ 4'6"
◯ 4'7"
◯ 4'8"
◯ 4'9"
◯ 4'10"
◯ 4'11"
◯ 5'0"
◯ 5'1"
◯ 5'2"
◯ 5'3"
◯ 5'4"
◯ 5'5"
◯ 5'6"
◯ 5'7"
◯ 5'8"
◯ 5'9"
◯ 5'10"
◯ 5'11"
◯ 6'0"
◯ 6'1"
◯ 6'2"
◯ 6'3"
◯ 6'4"
◯ 6'5"
◯ 6'6"
◯ 6'7"
◯ 6'8"
◯ 6'9"
◯ 6'10"
◯ 6'11"
◯ 7'0"
◯ 7'1"
◯ 7'2"
◯ 7'3"

Weight (lbs):

____

Do you have any underlying/chronic medical conditions

☐ Asthma
☐ Cancer (other than localized skin cancer)
☐ Chronic Kidney Disease
☐ COPD
☐ Coronary Artery Disease
☐ Diabetes
☐ Heart Failure
☐ High Blood Pressure
☐ Immunodeficiency (e.g., HIV/AIDS)
☐ Other
☐ None

Do you smoke cigarettes?

◯ Yes
◯ No

How many pack per day?

◯ less than half a pack per day
◯ 0.5 packs per day
◯ 1 pack per day
◯ 1.5 packs per day
◯ 2 packs per day
◯ more than 2 packs per day

Do you vape or use e-cigarettes?
◯ Yes
◯ No

How often do you vape?

◯ a few times per week
◯ a few times a day
◯ several times a day
◯ regularly, throughout most of the day

Do you have an immunocompromising condition or medication?
◯ Yes
◯ No

If so, what is the condition or medication/s?
____

Have you cared for a known COVID+ patient?
◯ Yes
◯ No

How many days were you in contact with a known positive COVID patient?
____

Did you perform any aerosol-generating procedures on the patient?
◯ Yes
◯ No

Which of the following (select all that apply):
☐ bagging
☐ BiPAP
☐ bronchoscopy
☐ Code Blue/CPR
☐ disconnection of ventilator circuit
☐ intubation or extubation
☐ open suctioning
☐ other

Please specify the procedure:
____

Have you cared for any PUI/Suspected COVID+ patients?
◯ Yes
◯ No
◯ Maybe

How many days were you in contact with a PUI / suspected COVID+ patient?
____

Did you perform any aerosol-generating procedures on the PUI?
◯ Yes
◯ No

Which of the following (select all that apply):

☐ bagging
☐ BiPAP
☐ bronchoscopy
☐ Code Blue/CPR
☐ disconnection of ventilator circuit
☐ intubation or extubation
☐ open suctioning
☐ other

Please specify the procedure: __________________________________

Do you have any known COVID+ community or home/family member contact?

◯ Yes
◯ No

If so, for how many days were you in contact with this person while they were symptomatic? __________________________________

Have you been furloughed from work due to suspected/known COVID+ ?

◯ Yes
◯ No

If so please provide the start date of the furlough: __________________________________

Have you had any new fever anytime this calendar year?

◯ Yes
◯ No

If so when did the fever start?

◯ 1-7 days ago
◯ 8-14 days ago
◯ 14-30 days ago
◯ 1-2 months ago
◯ 2-3 months ago
◯ >3 months ago

Please provide an approximate start date of this symptom __________________________________

Did you measure your highest temperature? ◯ Yes ◯ No

Measurement (Fahrenheit) __________________________________

Have you had any chills anytime this calendar year?

◯ Yes
◯ No

If so when did the chills start?

◯ 1-7 days ago
◯ 8-14 days ago
◯ 14-30 days ago
◯ 1-2 months ago
◯ 2-3 months ago
◯ >3 months ago

Please provide an approximate start date of this symptom __________________________________

Have you had any new cough anytime this calendar year?

◯ Yes
◯ No

If so when did the cough start?

◯ 1-7 days ago
◯ 8-14 days ago
◯ 14-30 days ago
◯ 1-2 months ago
◯ 2-3 months ago
◯ >3 months ago

Please provide an approximate start date of this symptom

____

Have you had difficulty breathing anytime this calendar year?

◯ Yes
◯ No

If so when did the difficulty breathing start?

◯ 1-7 days ago
◯ 8-14 days ago
◯ 14-30 days ago
◯ 1-2 months ago
◯ 2-3 months ago
◯ >3 months ago

Please provide an approximate start date of this symptom

____

Have you had any new runny nose, sinus congestion or sore throat anytime this calendar year?

◯ Yes
◯ No

If so when did the runny nose, sinus congestion or sore throat start?

◯ 1-7 days ago
◯ 8-14 days ago
◯ 14-30 days ago
◯ 1-2 months ago
◯ 2-3 months ago
◯ >3 months ago

Please provide an approximate start date of any of these symptoms

____

Have you had a loss of smell or taste anytime this calendar year?

◯ Yes
◯ No

If so when did the loss of smell or taste start?

◯ 1-7 days ago
◯ 8-14 days ago
◯ 14-30 days ago
◯ 1-2 months ago
◯ 2-3 months ago
◯ >3 months ago

Please provide an approximate start date of any of these symptoms

____

Have you had any new or unusual muscle aches anytime this calendar year?	◯ Yes<br>◯ No
If so when did the new or unusual muscle aches start?<br><br>◯ 1-7 days ago<br>◯ 8-14 days ago<br>◯ 14-30 days ago<br>◯ 1-2 months ago<br>◯ 2-3 months ago<br>◯ >3 months ago	
Please provide an approximate start date of this symptom	__________________________________
Have you had any new fatigue or tiredness anytime this calendar year?	◯ Yes<br>◯ No
If so when did the new fatigue or tiredness start?<br><br>◯ 1-7 days ago<br>◯ 8-14 days ago<br>◯ 14-30 days ago<br>◯ 1-2 months ago<br>◯ 2-3 months ago<br>◯ >3 months ago	
Please provide an approximate start date of this symptom	__________________________________
Have you ever been tested for COVID-19?	◯ Yes<br>◯ No
If so when were you tested?	__________________________________
What was your result?	◯ Positive<br>◯ Negative<br>◯ Pending
Were you told by your doctor that you could have COVID-19 but that testing was unavailable or not needed?	◯ Yes<br>◯ No
Where you hospitalized for COVID?	◯ Yes<br>◯ No
If so, on what date:	__________________________________
Were you treated with oxygen?	◯ Yes<br>◯ No
For how many days?	__________________________________

Were you treated in the ICU?	◯ Yes<br>◯ No
For how many days?	__________________________
Were you on a mechanical ventilator?	◯ Yes<br>◯ No
For how many days?	__________________________
Did you receive any of the following medications?	☐ hydroxychloroquine<br>☐ remdesivir<br>☐ tocilizumab<br>☐ steroids<br>☐ lopinavir/ritonavir<br>☐ antibiotics<br>☐ none of the above
